# Supplementary figures and images for: Gap junctions allow transfer of metabolites between germ cells and somatic cells to promote germ cell growth in the Drosophila ovary
Source: PLoS Biol. 2025 Feb 18;23(2):e3003045. doi: 10.1371/journal.pbio.3003045 (PMC11864552; doi:10.1371/journal.pbio.3003045)

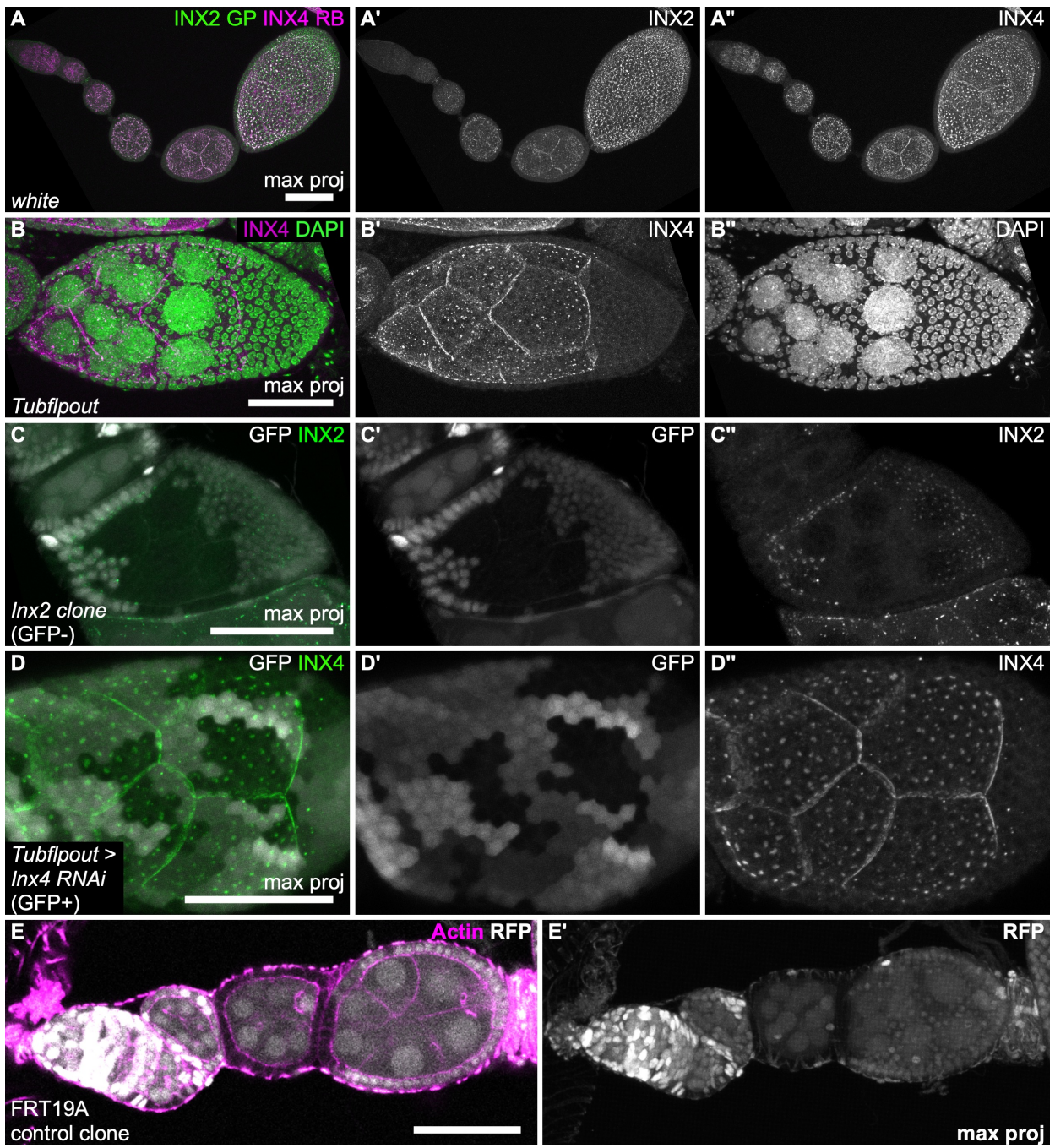

Supplement: S1 Fig — (A) Maximum intensity projection images of an ovariole after immunostaining for Inx2 (green in A, white in A′) and Inx4 (magenta in A, white in A″). Note the progressive increase of Inx2 expression. (B) Control stage 9 follicle (no clone) showing that GAP junction plaques are absent at oocyte-follicle cells interface. (C) Follicle containing a germline mutant clone for Inx2 (absence of GFP white in C and C′) and showing no growth defect and no impact on gap junction plaques while Inx2 staining (green in C, white in C″) is lost in the somatic clone observed on the same follicle. (D) Somatic Inx4 RNAi clone showing no impact on plaque formation (Inx4 staining green in C, white in C″). E) Control FRT19A RFP minus clone covering the whole epithelium of a follicle and showing no growth defect when compared to surrounding follicles. Scale bars 50 μm in A, 10 μm in all the other panels. (PDF) [file pbio.3003045.s001.pdf]

A

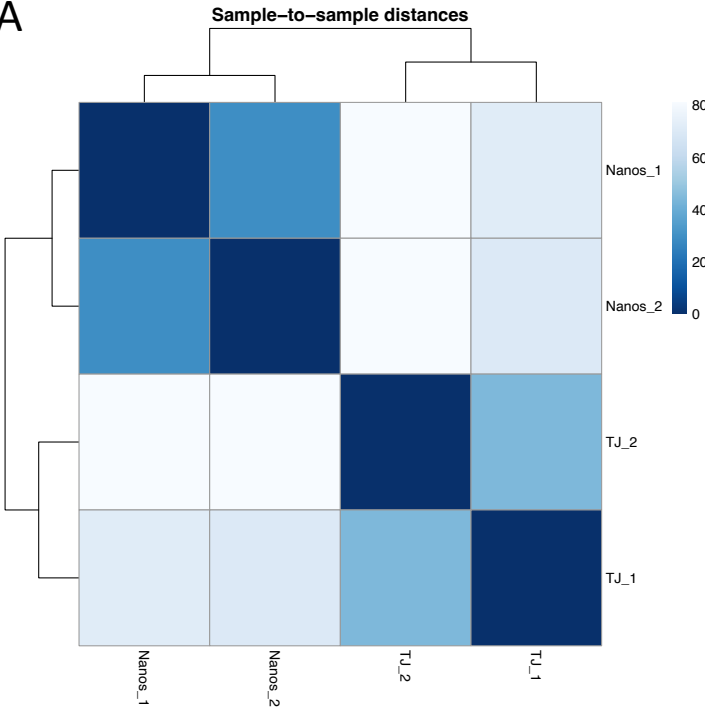

B

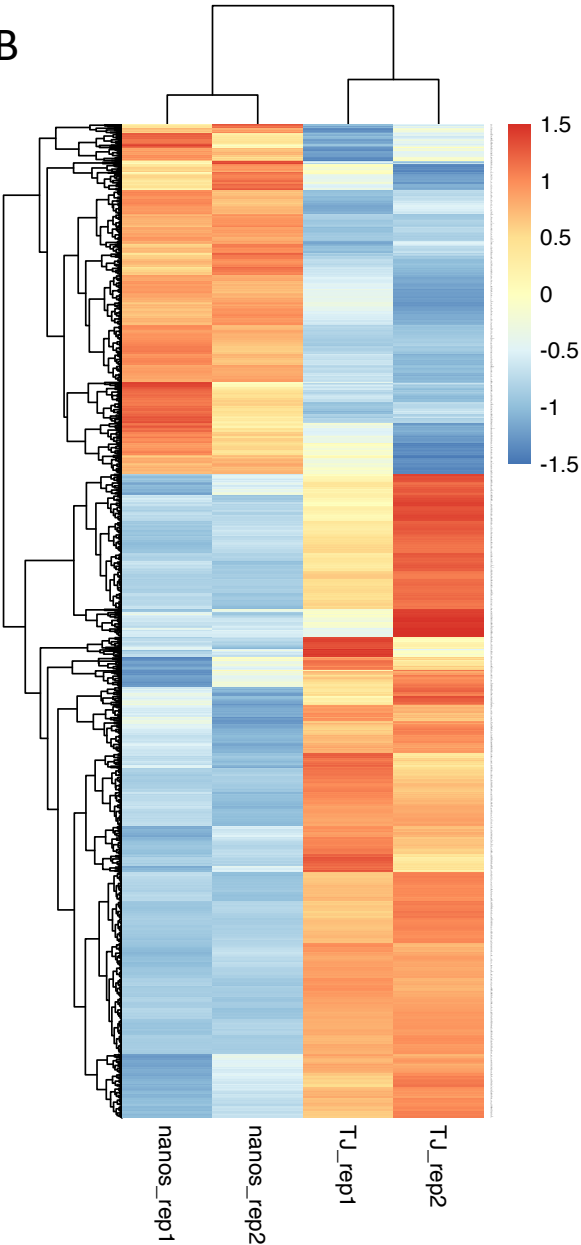

Supplement: S2 Fig — (A) Color-coded graph representing Spearman correlation coefficients between the different TRAP samples. (B) Z-score hierarchical clustering heat map visualization. Only significantly differentially expressed genes are reported. Highly expression correlation can be noticed across condition replicates. The raw data underlying this figure can be found in S1 Table. (PDF) [file pbio.3003045.s002.pdf]

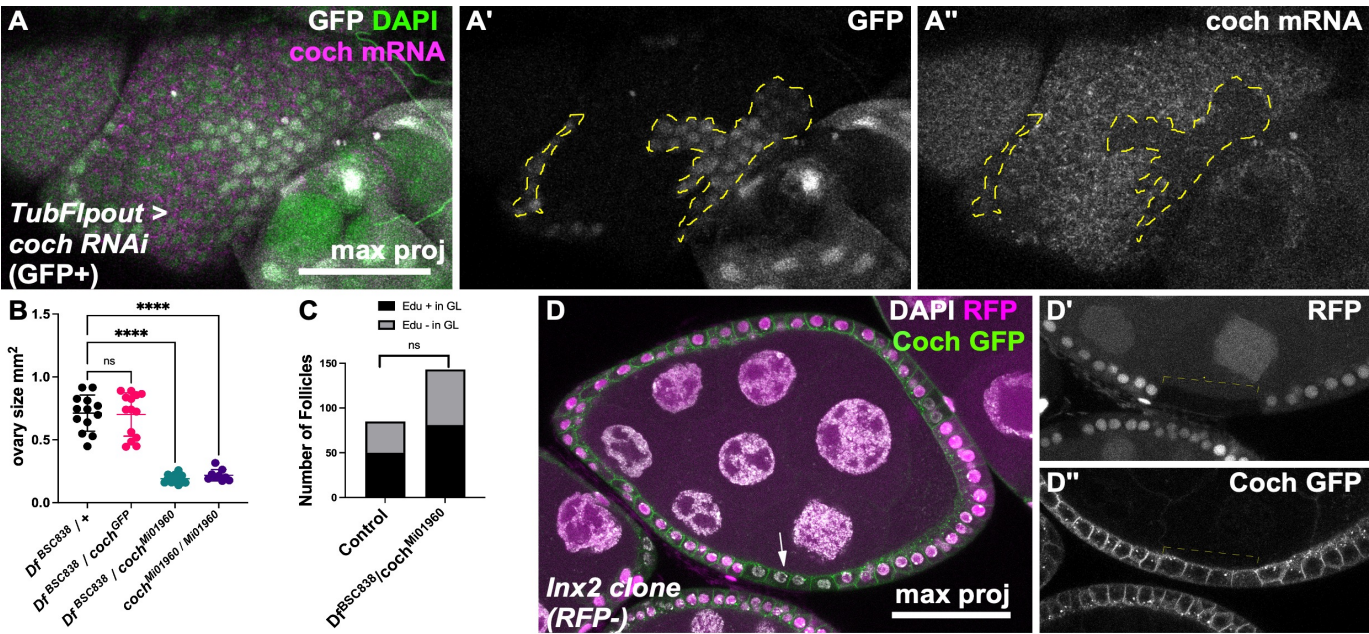

Supplement: S3 Fig — (A) RNAi clone against CG43693/coch stained by FISH against CG43693/coch showing its disappearance from these cells, confirming probe specificity and RNAi efficiency. (B) Quantification of ovary size (mm2) for the indicated genotypes (n = 13, 14, 12, 9, One-way ANOVA plus Dunnett’s multiple comparisons test). Data are the mean ± SD. ****p < 0.0001. (C) quantification of stage 1–8 follicles positive or negative for Edu in the germ cells (GL) (n = 85 for control and n = 143 for coch mutant flies, Fisher’s exact test). (D) Coch-GFP in a Inx2 mutant clone (RFP negative cells). Scale bars 10 μm. The raw data underlying this figure can be found in S1 Data. (PDF) [file pbio.3003045.s003.pdf]

A

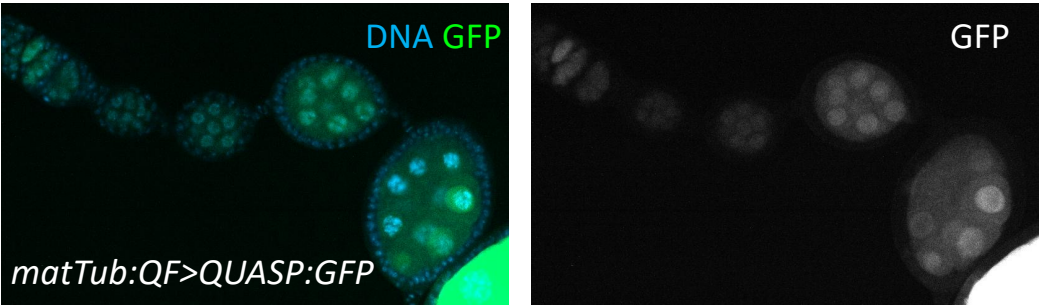

B

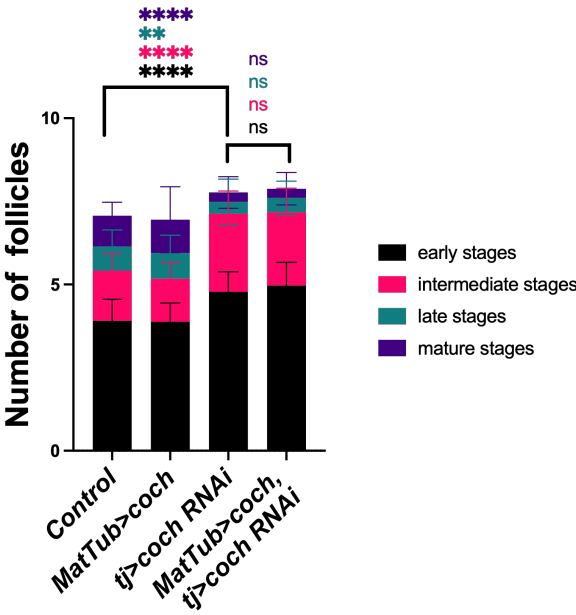

Supplement: S4 Fig — (A) Ovariole expressing a QUASP:GFP transgene under the control of the MatTub:QF driver. (B) Stage distribution per ovariole in indicated genotypes. We regrouped follicle stages in four categories: early: 1–6, intermediate: 7–9, late: 10–12, mature: 13–14 (n = 43 for controls and n = 95 for coch RNAi, n = 58 for qUAS:coch and n = 58 for cochRNAi, qUAS:coch, two-way ANOVA and Šídák’s multiple comparisons test). The raw data underlying this figure can be found in S1 Data. (PDF) [file pbio.3003045.s004.pdf]

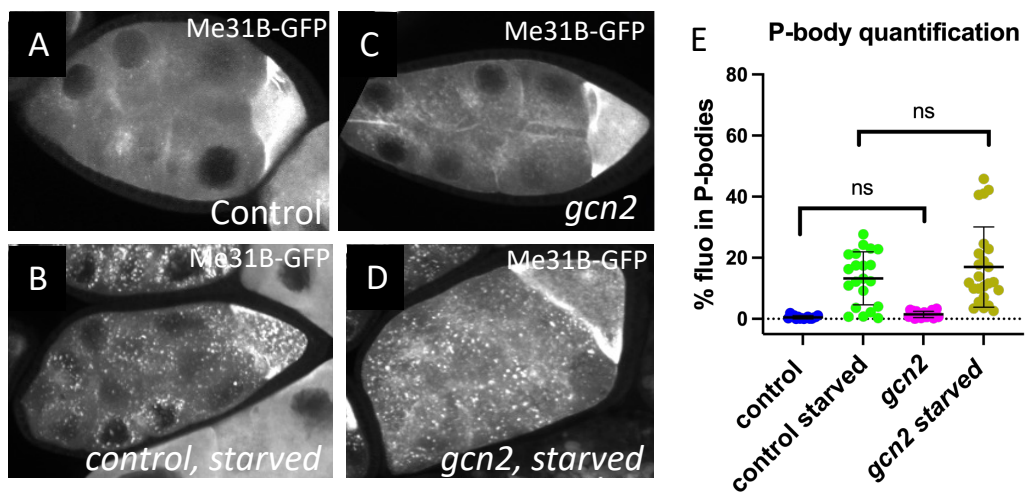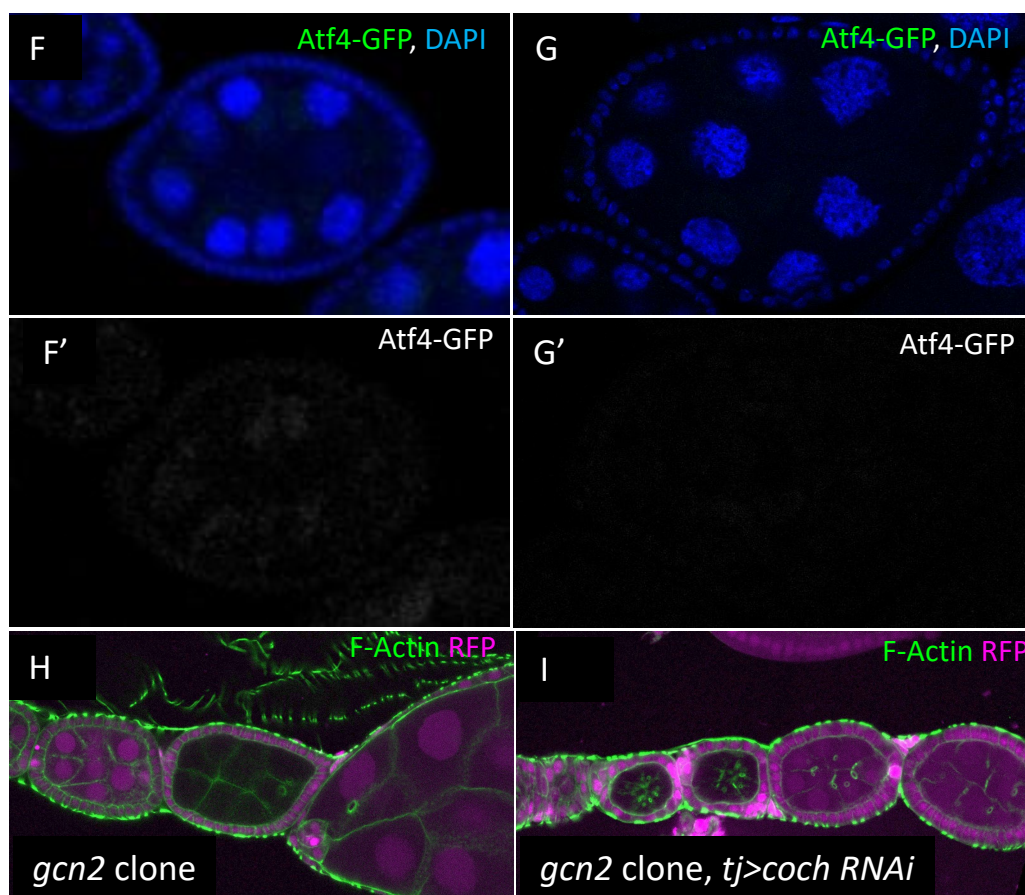

Supplement: S5 Fig — (A, B) Representative images of Me31B-BFP expression in stage 9 follicles from a: (A) control female, (B) starved control female, (C) gcn2 transheterozygous mutant female, and (D) protein starved gcn2 transheterozygous mutant female. (E) P-bodies quantification (fluorescence intensity) in follicles of the indicated genotypes and conditions. Data are the mean ± SD. (F, G) Absence of Atf4-GFP protein expression used as a read-out of GCN2 activity in (F) normal and (G) protein-starved conditions (green in F and G. white in F′ and G′). (H, I) Ovarioles with germline gcn2 mutant clones marked by the absence of RFP expression (magenta) and stained for F-actin (green) in (H) wild-type background and (I) in flies harboring coch RNAi in follicle cells. The raw data underlying this figure can be found in S1 Data. (PDF) [file pbio.3003045.s005.pdf]

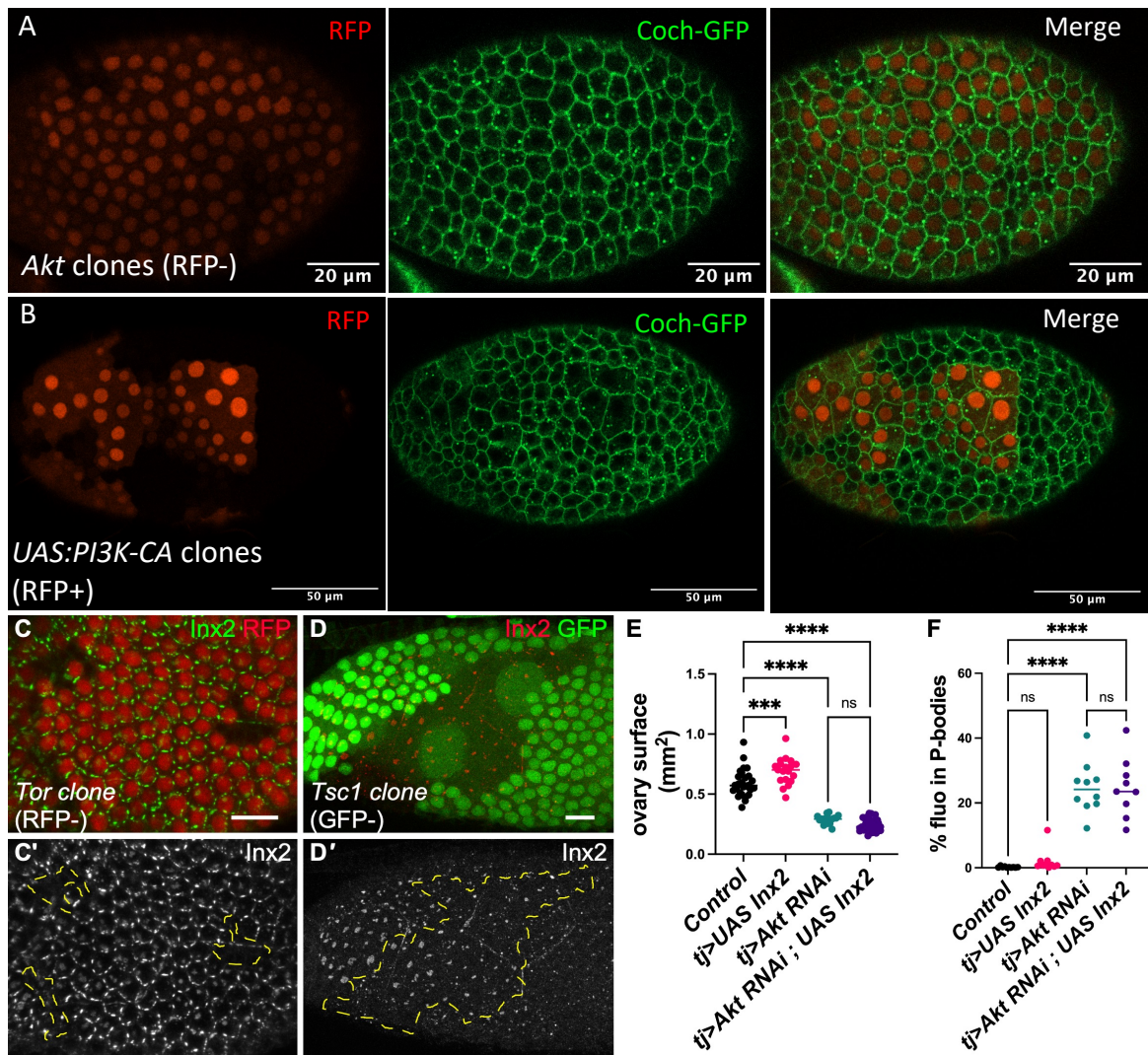

Supplement: S6 Fig — (A) Coch-GFP expressing follicle with akt mutant clones (RFP-negative cells). (B) Coch-GFP expressing follicle with flip-out clones that express a constitutively active form of PI3K (RFP- positive cells). (C, D) Inx2 staining in follicles containing a mutant clones for (C) Tor or (D) Tsc1. (E, F) quantification of (E) ovary size and (F) P-bodies in the indicated genotypes (E: n = 28, 19, 18, 32 and One-way ANOVA plus Tukey’s multiple comparisons test, F: n = 10, 10, 10, 9 Kruskal Wallis test plus Dunn’s multiple comparisons test). For all graphs, data are the mean ± SD, ***p < 0.001, ****p < 0.0001. The raw data underlying this figure can be found in S1 Data. (PDF) [file pbio.3003045.s006.pdf]
