# Supplementary material for: Gap junctions allow transfer of metabolites between germ cells and somatic cells to promote germ cell growth in the Drosophila ovary
Source: PLoS Biol. 2025 Feb 18;23(2):e3003045. doi: 10.1371/journal.pbio.3003045 (PMC11864552; doi:10.1371/journal.pbio.3003045)
Supplement: S2 Table — (DOCX) [file pbio.3003045.s008.docx]

| RESOURCE OR REAGENT | SOURCE | IDENTIFIER |
| --- | --- | --- |
| **Experimental models: Organisms/Strains** | | |
| tj:Gal4 : P{w+mW.hs=GawB}NP1624 | Kyoto Stock Center | FlyBase ID: |
|  | (DGRC) | FBti0034540; |
|  |  | DGRC: 104055 |
| FRT19A, Inx2^A^ | Bloomington | FlyBase ID: |
|  | Drosophila Stock | FBal0291304; |
|  | Center | BDSC: 54481 |
| FRT19A, Inx2^B^ | Bloomington | FlyBase ID: |
|  | Drosophila Stock | FBal0291305; |
|  | Center | BDSC: 52363 |
| FRT19A, Inx2^G0173a^ | Bloomington | FlyBase ID: |
|  | Drosophila Stock | FBal0151828; |
|  | Center | DGRC: 111858 |
| UAS :Inx2 RNAi (JF02446) | Bloomington | FlyBase ID: |
|  | Drosophila Stock | FBti0128670; |
|  | Center | BDSC: 29306 |
| UAS : Inx4 RNAi (GL00447) | Bloomington | FlyBase ID: |
|  | Drosophila Stock | FBtp0068601; |
|  | Center | BDSC: 35607 |
| nos:Gal4Vp16 | Bloomington | FlyBase ID: |
|  | Drosophila Stock | FBti0012410; |
|  | Center | BDSC: 4937 |
| MatTub :Gal4Vp16 (V2H) | Bloomington | FlyBase ID: |
|  | Drosophila Stock | FBti0016915; |
|  | Center | BDSC: 7062 |
| UASp-RPL10a-GFP | Junion G. | Nd |
| UAS : CG43693 RNAi (GLV21061) | Bloomington | FlyBase ID: |
|  | Drosophila Stock | FBti0144657; |
|  | Center | BDSC: 35696 |
| UAS : CG43693 RNAi (GL01304) | Bloomington | FlyBase ID: |
|  | Drosophila Stock | FBti0149385; |
|  | Center | BDSC: 41873 |
| CG43693/Coch ^MI01960^ : Mi{MIC}CG43693^MI01960^ | Bloomington | FlyBase ID: |
|  | Drosophila Stock | FBti0132690; |
|  | Center | BDSC: 34264 |
| Coch/CG43693-EGFP : CG43693 ^MI01960-GFSTF.0^ | This study |  |
| DF(3L)BSC838 | Bloomington | FlyBase ID: |
|  | Drosophila Stock | FBab0046056 |
|  | Center | BDSC: 29023 |
| Me31B-GFP: P{PTT-GB}me31B^CB05282^ | Bloomington | FlyBase ID: |
|  | Drosophila Stock | FBal0211797 |
|  | Center | BDSC: 51530 |
| UASz : eIF2a | This study |  |
| UASz : eIF2a-S51A | This study |  |
| UASz : eIF2a-S51D | This study |  |
| MatTub :QF | This study |  |
| QUASp :EGFP | This study |  |
| QUASp :Coch | This study |  |
| UAS :gcn2 sgRNA | Vienna Drosophila | VDRC: 341108 |
|  | Resource Center |  |
| nos:Gal4Vp16, ; UAS:Cas9 | Bloomington | BDSC: 54593 |
|  | Drosophila Stock |  |
|  | Center |  |
| FRT gnc2^CB20^ | This study, contains a 2n postion 878 (gg) on gcn2-RA transcript |  |
| FRT gcn2^CB14^ | This study, contains a 1n deletion at position 877 (a) and a 9n insertion (tttccgacc) on gcn2-RA transcript |  |
| 4E-BPintron-dsRed | H. Ryoo | FlyBase ID: |
|  |  | FBtp0116205 |
| ATF4-GFP : Mi{PT-GFSTF.1}crcMI02300-GFSTF.1 | Bloomington | FlyBase ID: |
|  | Drosophila Stock | FBal0314614; |
|  | Center | BDSC: 59608 |
| FRT40A, Pten^dj189^ | Pan D. | FlyBase ID: |
|  |  | FBal0117670 |
| FRT82B, akt^q^ | S. Cohen | Nd |
| UAS : akt RNAi (HM04007) | Bloomington | FlyBase ID: |
|  | Drosophila Stock | FBti0130466 |
|  | Center | BDSC 31701 |
| UAS : PI3K-CA  (Chromosome II) | This study, obtained by P-element mobilization of UAS:PI3K92E-CAAX (FBal0156077) |  |
| FRT40A, dTor^ΔP^ | Bloomington Drosophila Stock Center | FlyBase ID:  FBal0120586  BDSC 7014 |
| FRT82B, Tsc1^29^ | Pan D. | FlyBase ID:  FBal0123968 |
| UAS:inx2 | G. Tanentzapf |  |
| **Antibodies** | | |
| anti-Inx4, rabbit | G. Tanentzapf | janv-00 |
| anti-Inx2, Guinea Pig | G. Tanentzapf | 1/400 |
| anti-Inx2 rabbit | P. Phelan | janv-00 |
| anti-coracle mouse | DSHB | C615.16 1/100 |
| anti-mouse –Alexa 488 | Life technologies | A21202 1/1000 |
| anti-rabbit -Cy5, Donkey | Jackson Immunoresearch | 711-175-152 dilution 1/1000 |
| Anti-rabbit pig-Cy3, Donkey | Jackson Immunoresearch | 711-165-152 |
|  |  | dilution 1/1000 |
| anti-guinea pig-Cy5, Donkey | Jackson Immunoresearch | 703-175-155; dilution 1/1000 |
| Anti-guinea pig-Cy3, Donkey | Jackson Immunoresearch | 706-165-148 dilution 1/1000 |
| **Chemicals, Peptides, kits** | | |
| Phalloidin Atto-488, -550, -633 | Sigma | #49409, #19083, #68825 |
| DAPI | Sigma | 100 µg/ml D8417-1MG |
| EdU Click-iT™ Plus Alexa 555 | Thermo Fisher | C10638 |
| Ovation 1-16 droso Universal RNA-seq kit | Nugen | No longer distributed |
| **Software and Algorithms** | | |
| Fiji |  |  |
| Prism |  |  |
