# Supplementary material for: Gap junctions allow transfer of metabolites between germ cells and somatic cells to promote germ cell growth in the Drosophila ovary
Source: PLoS Biol. 2025 Feb 18;23(2):e3003045. doi: 10.1371/journal.pbio.3003045 (PMC11864552; doi:10.1371/journal.pbio.3003045)
Supplement: S3 Table — (DOCX) [file pbio.3003045.s009.docx]

**Table S3: Genotypes and specific conditions**

(h: hours; HS: heat-shock, GP : grape juice agar plate)

| **Figure** | **Genotype** | **Condition** |
| --- | --- | --- |
| Figure 1 B-E F    G,M  H,I  K,L  J | *w^1118^*  *y,w,hs:flp122; Act5C:FRT-Stop-FRT-Gal4, UAS:GFP , UAS:Inx2 RNAi*  *y,w,hs:flp122; Act5C:FRT-Stop-FRT-Gal4, UAS:GFP , UAS:Inx4 RNAi*  *FRT19A, Inx2 / FRT19A, Ubi:nlsRFP, hs:flp122*  *FRT19A, Inx2 / FRT19A, Ubi:nlsRFP, hs:flp122*  *tj:Gal4, tub:Gal80ts*  *tj:Gal4, tub:Gal80ts; UAS Inx2 RNAi* | 25°C  1HS, 4 days at 30°C  1HS, 4 days at 30°C  1HS, 10-12 days at 25°C  1HS, 2 days at 25°C  18°C, 14h at 30°C |
| Figure 2 A | *tj:Gal4 / UASp :Rpl10a-GFP*  *and*  *UASp :Rpl10a-GFP; nos:Gal4VP16* | 25°C |
| Figure 3 A  B  C  D  E,F  G,H  I,J  K,L | *tj:Gal4*  *tj:Gal4 / UAS: CG43693 (coch) RNAi ^GL01304^*  *tj:Gal4*  *tj:Gal4 / UAS: coch RNAi^GLV210161^*  *tj:Gal4 / UAS: coch RNAi^GL01304^*  *tj:Gal4*  *tj:Gal4 / UAS: coch RNAi^GLV210161^*  *w^1118^*  *coch-GFP*  *y,w,hs:flp122; FRT2A, coch^MI01960^/ FRT2A, Ubi:nlsRFP*  *y,w,hs:flp122; FRT2A, coch^MI01960^/ FRT2A, Ubi:nlsRFP* | 4 days at 30°C  4 days at 30°C  4 days at 30°C  4 days at 30°C  25°C  25°C  2HS, 8 days at 25°C  1HS, 2 days at 25°C |
| Figure 4 A,B,F,G  A,C,F,G  A,D,F;G  A,E,F,G  H | *tj:gal4 /+ ; MatTub:QF /+*  *tj:gal4 / QUASP:coch; MatTub:QF /+*  *tj:gal4/+ ; MatTub:QF / UAS: coch RNAi^GLV21061^*  *tj:gal4 / QUASP:coch; MatTub:QF/ UAS: coch RNAi^GLV21061^*  *tj:Gal4, Tub:Gal80ts,, Me31B-GFP/+; MatTub:QF/+*  *tj:Gal4, Tub:Gal80ts, Me31B-GFP/ QUASP:coch; MatTub:QF/+*  *tj:Gal4, Tub:Gal80ts, Me31B-GFP/+; MatTub:QF/ UAS: Inx2 RNAi*  *tj:Gal4, Tub:Gal80ts, Me31B-GFP/ QUASP:coch; MatTub:QF/UAS: Inx2 RNAi* | 4 days at 30°C  18°C, 48h at 30°C |
| Figure 5 A,C  B  D  E  F,K  G  H,K  I,L  J  L | *Me31B-GFP*  *Me31B-GFP*  *MatTub :Gal4Vp16, Me31B-GFP , UASz: eIF2a*  *MatTub :Gal4Vp16, Me31B-GFP, UASz: eIF2a-S51D*  *tj:gal4, Me31B-GFP /+*  *tj:gal4,Tub:Gal80^ts^,Me31B-GFP / UAS:Inx2 RNAi*  *tj:gal4, Me31B-GFP / UAS: coch RNAi^GLV21061^*  *MatTub :Gal4Vp16, Me31B-GFP / UAS: Inx4 RNAi*  *tj:gal4,Tub:Gal80^ts^,Me31B-GFP /+*  *tj:gal4, Tub:Gal80ts / QUASP:coch; MatTub:QF /+*  *tj:gal4, Tub:Gal80ts/+; MatTub:QF/ UAS: Inx2 RNAi*  *tj:gal4, Tub:Gal80ts/ QUASP:coch; MatTub:QF / UAS: Inx2 RNAi*  *MatTub :Gal4Vp16, Me31B-GFP/+* | 25°C  25°C, 14h on GP  25°C  25°C  2 days at 25 °C  18°C, 14h at 30°C  2 days at 25 °C  2 days at 25 °C  18°C, 14h at 30°C  2 days at 25°C |
| Figure 6 A,C  B,C  D  E,F    G  H | *tj:Gal4, Tub:Gal80^ts^ Me31B-GFP*  *tj:Gal4, Tub:Gal80^ts,^ Me31B-GFP / UAS: akt RNAi*  *y,w,hs:flp122; FRT82B, akt^q^ / FRT82B, Ubi:GFP*  *y,w,hs:flp122 ; FRT40A,Pten^dj189^ / FRT40A, Ubi:nlsRFP*  *y,w,hs:flp122, Tub:Gal4, UAS :GFP ; FRT40A,Pten^dj189^ / FRT40A, Tub :Gal80*  *y,w,hs:flp122, Tub:Gal4, UAS :GFP ; FRT40A,Pten^dj189^ / FRT40A, Tub :Gal80; UAS :Inx2 RNAi* | 2 days at 30°C  2 days at 30°C  1HS, 4 days at 25°C  1HS, 4 days at 25°C  1HS, 5 days at 25°C  1HS, 6 days at 25°C  1HS, 6 days at 25°C |
| Figure S1 A  B  C  D  E | *w^1118^*  *y,w,hs:flp122; Act5C:FRT-Stop-FRT-Gal4, UAS:GFP, UAS:Inx4 RNAi*  *FRT19A, Inx2 / FRT19A, Ubi:nlsRFP, hs:flp122*  *y,w,hs:flp122; Act5C:FRT-Stop-FRT-Gal4, UAS:GFP , UAS:Inx4 RNAi*  *FRT19A, Ubi:nlsRFP, hs:flp122 / FRT19A, Ubi:GFP* | 25°C  1HS, 5 days at 25°C  1HS, 5 days at 25°C  1HS, 5 days at 25°C |
| Figure S3 A  B  C  D | *y,w,hs:flp122; Act5C:FRT-Stop-FRT-Gal4, UAS:GFP / UAS: coch RNAi*  *DF(3L)BSC838/+*  *DF(3L)BSC838 / coch^MI01960^*  *coch^MI01960^ / coch^MI01960^*  *DF(3L)BSC838 / coch-GFP*  *w1118*  *DF(3L)BSC838 / coch^MI01960^*  *FRT19A, Inx2 / FRT19A, Ubi:nlsRFP, hs:flp122 ;; coch-GFP* | 1HS, 3 days at 30°C  25°C  25°C  1HS, 4 days at 25°C |
| Figure S4 A  B | *MatTub:QF / QUASP:GFP*  *tj:gal4 /+ ; MatTub:QF /+*  *tj:gal4 / QUASP:coch; MatTub:QF /+*  *tj:gal4/+ ; MatTub:QF / UAS: coch RNAi^GLV21061^*  *tj:gal4 / QUASP:coch; MatTub:QF/ UAS: coch RNAi^GLV21061^* | 25°C  4 days at 30°C |
| Figure S5 A,C  B,C  D,E  H  I | *Me31B-GFP*  *Me31B-GFP, gcn2^CB20^/ gcn2^CB14^*  *ATF4-GFP*  *y,w,hs:flp122 ; FRT82B,  gcn2^CB14^ / FRT82B, Ubi:nlsRFP*  *y,w,hs:flp122 ; tj :Gal4 ; UAS: coch RNAi^GL01304^, FRT82B,  gcn2^CB14^ / FRT82B RFP* | 25°C  25°C  25°C  1HS, 5 days at 30°C  1HS, 5 days at 30°C |
| Figure S6 A  B  C  D  E,F | *y,w,hs:flp122 ; coch-GFP, FRT82B, akt^q^ / FRT82B, Ubi:nlsRFP*  *y,w, hsflp122; Act5C:FRT-STOP-FRTGal4; UAS:RFP/ UAS:PI3K-CAAX ; coch-GFP*  *y,w,hs:flp122, FRT40A, ubi :nlsRFP / FRT40A, dTor*  *y,w,hs:flp122, FRT82B, Ubi:nlsGFP/ FRT82B, tsc1^29^*  *tj:Gal4, Tub:Gal80ts, Me31B-GFP/+;*  *tj:Gal4, Tub:Gal80ts, Me31B-GFP/ UAS Inx2*  *tj:Gal4, Tub:Gal80ts, Me31B-GFP/+; UAS: Akt RNAi/+*  *tj:Gal4, Tub:Gal80ts, Me31B-GFP/ UAS Inx2; UAS: Akt RNAi/* | 1HS, 5 days at 25°C  1HS, 5 days at 25°C  1HS, 4 days at 25°C  1HS, 5 days at 25°C  2 days at 30°C |
