# Supplementary material for: Gap junctions allow transfer of metabolites between germ cells and somatic cells to promote germ cell growth in the Drosophila ovary
Source: PLoS Biol. 2025 Feb 18;23(2):e3003045. doi: 10.1371/journal.pbio.3003045 (PMC11864552; doi:10.1371/journal.pbio.3003045)
Supplement: Attachment — Submitted filename: Response to the Reviewers .pdf [file pbio.3003045.s011.pdf]

First of all, we would like to thank the reviewers for recognizing the wide interest, originality and quality of our work. We also understand their comments and we tried our best to improve our article in the time we had.
In this document our replies are in blue, the cited text in purple, and where there is a mix of old and new text, the latter is underlined.

Rev. 1:

The manuscript by Vachias et al. examines a potential role for gap junctions in coordinating growth between somatic and germline cells during oogenesis by allowing transfer of metabolites. Previous studies in the mouse showed that gap junctions between somatic granulosa cells and the oocyte are required for oocyte growth and that metabolic coupling between these cell types is required for oocyte growth. Growth of the somatic and germline cells within the Drosophila ovarian follicle is also coordinated as shown by this group and others. Gap junctions have been visualized between the somatic follicle cells and between follicle cells and the ooctye/nurse cell complex and, although not acknowledged here, have been shown to permit communication between the follicle cells (Ref 28). However, whether they also coordinate somatic and germline growth in the Drosophila ovary and what metabolites are transferred isn't known. Although visualizing metabolite transport is not feasible in this system, the authors use alternative and clever approaches to provide evidence that the Drosophila ovarian follicle cells influence germline growth and that they do this at least in part by supplying amino acids whose transporters are not expressed in the germline. The data are convincing and for the most part, the conclusions are well supported. As discussed below, the assertions that gap junctions coordinate growth and that gap junctions ultimately regulate translation in the germline are less convincing. The work is strong enough to rest on the data provided, so the authors simply need to temper these assertions.

The authors show that heteromeric gap junctions form between the somatic and germline cells in the Drosophila ovary whereas homomeric junctions form between follicle cells. Using RNAi and genetic mutations, the authors show convincingly that gap junctions are indeed required for germline growth, but not for follicle cell growth. The finding that they are not required for follicle cell growth indicates that they nonautonomously influence germline growth, not that they coordinate follicle cell and germline growth (see the first concern above).

We agree that gap junctions *per se* do not coordinate the growth of somatic and germline tissues, as they do not regulate somatic growth. However, the fact that PI3K pathway controls both somatic growth and gap junction assembly illustrates this idea of coordination. Our previous work deeply analyzed the role of the PI3K pathway in the somatic cells to control both somatic growth and germline growth in such a way that both stay coordinated (Vachias et al, 2014). Our new findings on gap junction role and their control by the PI3K pathway come as a mechanistical explanation of this coordination. We tried to make this point clearer in the current version of manuscript by :

- Adding few words in the abstract : "Finally, in somatic cells, innexin2 expression and gap junction assembly are regulated by the insulin receptor/PI3K kinase pathway, <u>linking the growth of the two tissues."</u>
- changing the related subheading in the results
- by modifying the discussion with following sentences :

"We found that Inx2 expression and the subsequent formation of plaques were strongly regulated by the InR/PI3K pathway in a cell autonomous manner, <u>providing an effective mechanism to link metabolic flow with growth systemic signals. Since InR/PI3K pathway activity in the follicle cells controls both somatic growth and germline growth ensuring their coordination, its impact on gap junctions likely participates in this coordination</u> "

To identify metabolic pathways that use these gap junctions, they cleverly isolate mRNAs that are preferentially translated in follicle cells over the germline using TRAP. The data revealed differential production of proteins involved in amino acid synthesis or import. Although there are multiple ways to interpret this result, the authors focus the remainder of the study trying to support the idea that amino acids may be taken up by follicle cells and transported to the germline through gap junctions. RNAi knockdown of one of the genes identified, CG43693 (now named coch), a member of the SLC36 family of amino acid transporters, produced an ovary growth defect and both RNA and protein expression appear specific to the follicle cells. The authors nicely combine the QUAS and UAS systems and show that ectopic expression of coch in the germline can compensate for knockdown in the follicle cells. This supports the idea that the germline requires the activity of coch and amino-acid transport and because

coch normally only functions in the follicle cells. Although it would be ideal to have more direct evidence, the authors argue reasonably that the data support direct metabolic exchange between somatic and germline cells.

In the remainder of the manuscript, the authors try to connect metabolic transport to the translational state of the oocyte, which I find to be much less convincing. It is clear that P bodies do form when gap junctions are eliminated or when coch is knocked down, but whether this relates to depletion of amino acids is not clear. Me31B containing P bodies form/enlarge under many different stress conditions and it seems more likely that they are a consequence of metabolic stress (from any one of many possible metabolites not considered here) rather than a specific mechanism to stop translation. Therefore, the statement that although the statement "P-body formation is linked to germ cell growth control by gap junctions and amino acid import in follicle cells" the authors should make it clear that P-body formation may be a downstream consequence rather than direct effector of this growth control.

It is indeed true that we do not know the mechanism leading to P-bodies and that it might be indirect as we have not been able to directly link their formation with amino acids and GCN2 activity. However, reading your comments help us to realize that we did not emphasize enough some our results. Especially, mimicking eif2alpha phosphorylation, which is known to be sufficient to block translation and induce P-bodies, is also sufficient to block germline growth. We therefore tried to better explain this point but also moderate our conclusions:
In the abstract : "Moreover, affecting either gap junctions or the import of some amino acids in somatic cells induces P-bodies in the germ cells, a feature <u>usually</u> associated with an arrest of translation".
In the results : "P-body formation <u>is usually induced by inhibitory phosphorylation of the eukaryotic translation initiation factor 2 subunit alpha (eIF2α) on serine 51 (S51), an event also known to cause a translation arrest (</u>Riggs et al, 2020)… Importantly, eIF2α-S51D overexpression was sufficient to completely block germline growth, suggesting a ~~direct~~ link between P-body formation and growth control (Fig. 5E)… *Coch* and *Inx2* knockdown strongly induced the formation of P-bodies, suggesting that the observed growth limitation was linked to their formation and a <u>potential</u> arrest of translation (Fig 5F-I)…. Altogether these data indicate that although the two well-established pathways involved in amino acid sensing do not seem implicated, P-body formation <u>acts as a metabolic stress sensor linked to the control of</u> germ cell growth by gap junctions and the import of some amino acids in follicle cells."
In the discussion :
Moreover, <u>direct genetic induction of P-bodies in the germline is sufficient to block germline growth</u>, and both defective gap junction or amino-acid import in somatic cells induces P-bodies in the germ cells, a feature <u>usually</u> associated with an arrest of translation. <u>However, their effect is not mediated by GCN2 and it is therefore unclear by which mechanism P-bodies are induced in such a situation.</u> Thus, <u>the most plausible explanation</u> for all our data is a model in which gap junctions allow the transfer from somatic cells to germ cells of metabolites, such as amino acids, that germ cells cannot directly produce or import, thereby promoting germ cell growth through translation control.

Specific issues:

1) Why are the follicle cells unaffected in coch mutants or when gap junctions are disrupted throughout the epithelium (so there is no exchange between follicle cells)?

It is unclear why coch is not required in follicle cells. One potential explanation is that the overall increase in follicular epithelium volume is significantly less than that of the germline cyst. Indeed, considering that the thickness of the epithelium varies little from one stage to another, the growth of the epithelium is proportional to the square of its dimensions, whereas for the germline cyst, growth is proportional to the cube. Therefore, the follicular epithelium may be much less sensitive to metabolite availability than the germline. A sentence has been added in the discussion :
"Second, female germ cells undergo massive growth and there is a non-linear relation between volume and cell surface increases. Consequently, surface exchange with the extracellular medium might not allow sufficient metabolic import and may require the support of follicle cells. This hypothesis was proposed to explain gap junction requirement for mammal oocyte growth (Su et al, 2009). <u>It could also explain why germline growth but not somatic growth is sensitive to the loss of *coch*, as follicular epithelium has a relatively constant height and thus increases much less in volume than the germline."</u>

Regarding GAP junctions, our interpretation is that they are unidirectionally required for growth, meaning the germline provides no essential metabolite for follicle growth. Moreover, they are other innexins expressed in follicle cells an found in between follicle cells (but not between germline and follicle cells) that may mask a role of gap junction in between follicle cells. This point is now evoked in the manuscript :
"Of notice, follicle cells express other innexins at their lateral domain, suggesting that functional gap function in between follicle cell may still exist in absence of Inx2 and may mask a potential communication in between these cells influencing their growth (Bohrmann et al, 2008). Nonetheless, these results suggest that Inx2-dependent gap junctions are specifically required for germline growth."

2) The authors refer to amino acid transport generically but with coch knockdown/mutation, only a small subset of amino acids is not transported. The authors should be careful about implying that all amino acid transport is blocked. Moreover, transport from hemolymph to the oocyte can occur directly up to stage 8, and the authors show that some transporters are expressed in the oocyte. Finally, the 5-fold enrichment threshold used to designate gene expression as specific to the follicle cells doesn't mean that there is no expression in the oocyte and without RNAi/knockdown experiments as performed for coch it is premature to conclude that there is no expression of a particular transporter in the germline.

We indeed do not claim that the transport of all amino acids is affected. We tried to make this point clearer thorough the manuscript, unless when affecting too much sentence readability. We also tuned down our description of Coch profile based on TRAP, mentioning a strong enrichment in the follicle cells, and we start speaking about specific somatic expression only from the FISH experiment.

3) It would be nice to know what aspects of growth are affected in the germline. Other effectors of growth like myc affect nurse cell endoreplication (doi: 10.1242/dev.00932). Effects endoreplication are relatively easy to assay.

We know include quantification of the proportion of Edu-positive germline cysts in *Inx2* RNAi (Fig 1J) and *coch* mutants (Fig S3C). While Inx2 kd induces a significant reduction of the proportion of cyst undergoing endoreplication, this is not the case for *coch* mutant follicles. One possible interpretation is that temporally controlled Inx2 kd induces an acute and strong arrest of germline growth, associated with a cell cycle arrest, while *coch* has a milder effect that may lag each cell cycle phase but then do not significantly affect their respective proportion. In agreement with this idea, when somatic clones for *coch* induce smaller follicles, as shown on Fig3I, it is also associated with smaller nurse cell nuclei, that strongly suggests a delay in the cell progression through the rounds of endoreplication. We therefore propose this interpretation in the discussion.

4) Knockdown of other genes identified in the TRAP experiment did not result in phenotypes and the authors suggest that there may be multiple levels of redundancy. Do they have evidence that the RNAi actually worked?

We have not attempted systematic verification of RNAi efficiency and it is indeed therefore possible that we missed required genes, though we tested all the available RNAi lines at that time. We added the following sentence. "Moreover, although all the available RNAi lines for these genes were tested, we could not exclude that some were not efficient enough."

5) Figure 3D and E - the resolution is too low in these figures. At the resolution shown, the RNA looks like it is cortical, which makes the result questionable.

Coch mRNA is indeed enriched at the apical side of the follicle cells. This data is actually not surprising as it is the case for many different mRNAs in this tissue (Jambor et al, Elife 2015). Actually, looking again at this paper when writing these lines, we realized that they also found an apical localization for CG43693 mRNA. A sentence explains now this point : "This mRNA was enriched at the apical side of the follicle cell, fitting with a previous observation that many mRNAs, including this one, are localized at this domain in follicle cells (Jambor et al, 2015)."
We also changed the resolution of Fig3E.

6) For the genes whose translation is limited to the follicle cells, it would be interesting to know if they are only transcribed there and not in the nurse cells, or if their translation is controlled so that they are only translated in the follicle cells. Are there RNA seq data available that could be used to answer this?

The example of *coch* suggests that they are only transcribed in follicle cells. Interrogating the Fly Single Cell Atlas indicate that they are all detected in a higher proportion of “ovariole somatic cells” than “female germ cells”, suggesting that the differences we observed in TRAP experiments reflects differences in mRNA expression level and are not explained by translation regulation. It is now mentioned in the results : “Interrogating the Fly Cell Atlas for the 18 genes involved in amino acid synthesis or transport and enriched in the soma in the TRAP experiments indicated that they are all detected in a higher proportion of “ovariole somatic cells” than “female germ cells”. It suggests that the differences observed in the translatome of soma and germline are already true at the level of their transcriptomes.”

7) The hierarchical clustering shown in Fig. S1 is not a good way to show reproducibility of results. A graph with Pearson correlation coefficients is preferable.

In this specific case, Spearman correlation seems to us more appropriate. A graph with these coefficients is now provided Figure S2A.

8) Some figures are labeled with CG43693 and some with coch (even before the name is defined). Best to be consistent throughout or to use CG43693 in the early figures and coch after the name is defined.

We decided to name it “coch” sooner in the manuscript, though the explication for its name comes a bit later in the article. We hope it makes it easier to read.

Rev. 2: Mayu Inaba –

The article by Vachias et al demonstrates that gap junctions participate in metabolite transfer from somatic follicle cells to germline to support oogenesis. Using polysome-seq, which can reliably detect cell-type specific gene expression, authors identified an amino acid transporter, coch, specifically expressed in follicle cells but required for growth of germ cells. Coch knockdown phenotype in somatic FCs is rescued by ectopic expression of Coch in germline, supporting the proposed idea of metabolic exchange between these cell types. Overall, the authors perform well thought out experiments, which support a novel mechanism how somatic epithelia support oogenesis via sending amino acid through gap junctions. There are, however, some points to be consider worth revising for the overall improvement of this manuscript.

Major points:

1: As authors stated, metabolic coupling between soma and germline is solely supported by experiments shown in Fig4, in which they show coch expression in the germline can bypass its silencing in the soma. I feel this experiment needs to be done with better methods, such as checking frequency/distribution of stages of egg chambers.

We analyzed stage distribution in these different genetic contexts by classifying follicles in four groups. The loss of Coch in follicle cells clearly induced a depletion of mature stages and an accumulation of young and intermediate stages, that are probably delayed in their growth. These data have been included on Fig3, as asked by R#3. In the experiment of rescue by the germinal expression of *coch*, we could not point out the rescue effect on this distribution (Fig S4). Why the stage distribution appears less sensitive to detect the rescue is not fully clear. One potential explanation is that the presence of few more mature eggs may not be seen in the distribution but may impact ovary volume. To go further, we also counted the number of laid eggs in each condition (new Fig 4G). We observed significant tendencies confirming previous results based on ovary size. Overall, the results confirm the rescue but it appears a bit more subtitle than only based on ovary size, and we therefore moderated our conclusions:
In the abstract :
Its ectopic expression in the germline can <u>partially</u> compensate for its absence in somatic cells.
In the results :
<u>However, such rescue is not quantitatively observed in the stage distribution, suggesting that the latter is less sensitive than ovary size to probe growth modification (Fig. S4B). We also quantified egg laying in these different genetic backgrounds. This experiment confirmed both the slight deleterious effect of germline *coch* expression but also its ability to rescue its knockdown in the follicle cells (Fig. 4G).</u> Altogether, these genetic data demonstrated that the requirement of coch expression in follicular cells for germline growth can be <u>partially</u> compensated by its germline expression, suggesting a direct metabolic exchange between these cell types.
In the discussion
Accordingly, our results showing a <u>partial</u> rescue of *coch* absence in the soma by its ectopic expression in the germline imply that its solutes can reach the oocyte.

2. Can inx4 RNAi in germline (or inx2 in soma) phenotypes be suppressed by expression of Coch in germline? I understand that there should be many other materials to be transferred through gap junctions, but I wonder if those amino acids could be essential for oogenesis, if so the rescue may occur even partially. On the other note, p-body formation seems to be a good lead-out and I wonder if authors can better quantify non-cell autonomy using this.

We really thank R#2 and R#3 for this suggestion. We attempted this experiment and observed a tendency towards a partial rescue of ovary size (Fig. 4H) : To further explore this idea, we attempted similar experiments between the ectopic expression of *coch* in the germline and the *Inx2* knockdown. Such a condition tended to rescue the ovary size of *Inx2* knockdown (Fig. 4H). Interestingly, this result fits with a model in which the solutes imported via Coch in the somatic cells normally reach the germline through Inx2-dependent gap junctions.

Moreover, fitting with an idea of a partial rescue, we observed a significant decrease of p-bodies in such a genetic combination compared to *Inx2* kd alone. Of notice, because P-bodies were not mentioned before, these important results are now shown on Fig. 5 and are described as follow:
"Finally, since ectopic *coch* expression in the germline tended to rescue ovary size in somatic *Inx2* knockdowns, we asked whether a similar effect could be observed on P-bodies. Interestingly, we found a significant reduction

in P-bodies compared to *Inx2* knockdown (Fig 5L). These results provide another indirect argument in favour of a model based on the exchange of solutes across gap junction that are initially imported by Coch in the somatic cells."

3. Can authors visualize inx2/4 patch in better resolution? In later vitelline membrane localize between germline and soma and I wonder if gap junctions are formed between microvilli.

There is no more gap junction at the interface between follicle cells and the oocyte from the stage where microvilli form (Stage 9). We added a sentence to precise this point, and an illustration of the corresponding stage (Fig. S1B): "However, plaques tended to disappear specifically from the oocyte cortex around stage 8 and were completely absent at this position from stage 9 onwards (Fig. S1B)."

4. The logic behind the experiments checking TOR and GCN2 function (Figure S5, line 256 to 270) is difficult to follow. It would be helpful for readers if authors can revise this portion. Especially, it is unclear why conclusion differ between previous studies and the current study.

We tried to better introduce and conclude this paragraph :
"We hypothesized that the non-cell autonomous effect of *coch* and *Inx2* RNAi on the formation of P-bodies could a be consequence of the deprivation for some amino acids. P-bodies or stress granules formation in response to a lack of some amino acids can occur independently or dependently of eIF2α S51 phosphorylation. In the first case, which is not the most frequently described, it is associated with the repression of the TOR pathway. However, induction of Tor RNAi in the fly germline does not induce P-body formation, excluding this possibility (Burn et al, 2015). In the second case, eIF2α is phosphorylated by GCN2 that acts as an indirect sensor of amino acid availability. We generated null mutant alleles for *gcn2* that were homozygous viable, as similar alleles described during the course of this project. Protein deprivation in *gcn2* transheterozygous females still led to P-body formation in the germ cells (Fig. S5A-E). Moreover, an indirect reporter of GCN2 activity and eIF2 αS51 phosphorylation (ATF4-GFP) showed no signal in the germline of wild-type flies, even after protein deprivation (Fig. S5F-G). Lastly, we tested more directly gcn2 role in the germline when amino acid import in follicle cells is impaired (tj>cochRNAi). However, gcn2 mutant germline clones did not increase the growth rate of wild-type and coch RNAi follicles (Fig. S5H,I). These results strongly argued against the implication of GCN2 as a germ cell growth repressor when the availability of the amino acids provided by Coch is reduced. Thus, being independent of TOR and GCN2, the mechanism leading to P-bodies formation in the female germline seems unusual and will require further investigation."

5. Can inR effect or starvation on p body formation be cancelled by inx2 overexpression

We attempted Inx2 over-expression in conditions of *akt* RNAi and observed no rescue nor on ovary size nor on p-bodies formation, suggesting that Inx2 is not sufficient to explain the effect of PI3K pathway activity in follicle cells for its non-cell autonomous effect on germline cell growth. These data are now added into Fig. S6 and mentioned in the result as follow :
"We also tested whether *Inx2* overexpression could be sufficient to rescue the knockdown of the InR/PI3K pathway in the somatic cells. In this genetic combination, we observed neither an increase in ovary size nor a decrease in P-bodies compared to the *akt* knockdown. Thus, the control of Inx2 expression is not sufficient to explain the non cell-autonomous effect on germline growth of the InR/PI3K pathway in the follicle cells."
And in the discussion :
Finally, the fact that Inx2 expression in follicle cells is not sufficient to explain the non cell-autonomous effect on germline growth of the InR/PI3K pathway in the follicle cells, strongly suggests that this pathway controls other genes involved in the exchanges between the two tissues.

Minor points:
1. Quantification method of p-bodies are showing fluorescent intensity per detected p-body by taking minimum intensity of 1.75 compared with neighbouring pixels? If so, how authors quantified control?

We are not sure to fully understand this comment. As explained in the method section, our P-body quantification is not based on fluorescent intensity per detected p-body but rather by 1) automatic detection of P-bodies as defined

by at least five adjacent pixels with an intensity 1.75 higher than the mean intensity in the nurse cells 2) quantification of total fluorescence contained in all these p-bodies as a fraction of the total intensity in the nurse cells.

2. fig 6D pattern of inx2 staining of surrounding wt FCs looks different from others.

In the wildtype situation, the Inx2 pattern is actually quite complicated depending on the stage, the position on the follicle. For instance, apical plaques are usually larger in the anterior part around stage 6-8 as you might see it on fig 1. Moreover, levels of Inx2 strongly increase with the stage as shown on Fig. S1A and apical plaques are less evident to detect in early stages in the wildtype situation. Moreover, coming back to the Fig 6D, Inx2 levels are so high in *pten* mutant cells that we had to tune down the detection level to avoid saturation. However, you can still see the apical plaques in wildtype cells, especially in the anterior part.
We modified the following sentence in the initial description of the Inx2 profile :
As previously described, we detected Inx2 and Inx4 forming plaques at the interface between germ and somatic cells, these plaques often being larger in follicle anterior part (Tazuke et al, 2002; Bohrmann et al, 2008; Sahu et al, 2021).

Rev. 3:

The manuscript by Vachias et al have used the Drosophila oogenesis model to demonstrate that growth of the germline cells is dependent on the overlying somatic follicle cells. Employing classical genetics and by examining gap junction proteins, the authors claim that the communication through the gap junctions (Inx2 - Inx4) across soma and germline is critical for the growth of germ cells during oogenesis. In an effort to identify the nature of the molecule that may be passing through the Inx2-Inx4 heteromeric channel, the authors undertook a translatomic approach and narrowed down on a predicted amino acid transporter, CG43693. By depleting CG43693 function the authors claim that CG43693 is functioning in the follicle cells to support the germline growth. Finally by employing immunohistochemistry and epistasis experiments authors propose Inx2 functions down stream of Insulin signaling/ PI3K pathway in the follicle cells to facilitate germline growth. Overall the model proposed by the authors is quite novel but the primary claim needs further validation before it can be accepted. My comments are listed below.

Major comments:

1. The genetic evidence provided here doesn't unequivocally support the proposed model that Inx2- Inx4 mediated amino acid exchange between somatic cells and germline cells supports oocyte growth. This needs to be demonstrated more convincingly.

We understand this general comment. We hope that the new version of the article will be more convincing. Unfortunately, some of the points were impossible to answer with the tools and time we had. We have also adjusted some of our conclusions, as suggested by the other two reviewers.

2. The function of CG43693 gene needs to be validated before it can be accepted as an as an amino acid transporter in the proposed model.

It is true that it is not formally demonstrated that coch/CG43693 is an amino acid transporter. However, deep phylogenetic exploration clearly demonstrated that this gene encodes a protein of the SLC36 transporter family, all of them being well demonstrated amino acid transporters, though with different specificities. Determining this specificity is, of course, of interest but seems to us behind the scope of this paper as it cannot be easily determined. Classical experiments for amino acid specificity determination of such ion-coupled transporters usually implies heterologous expression in xenopus oocytes and uses voltage-clamp techniques under controlled conditions. We adjusted the text and mentioned Coch as a “putative” amino acid transporter throughout the article.

3. The aspect that Inx2- Inx4 colocalization is dependent on each other in developing egg chambers is a repeat of what has been shown by Sahu et al in 2021. How does adding similar data help in Fig 1.

We initially performed these experiments before this publication and we agree that they now come partially as a confirmation of the nice work by Sahu et al, which was mentioned in our article. However, in this latter work the interdependence between inx2 and Inx4 was shown only in one direction (i.e. Inx2 required for Inx4 localization). For instance, the fact that in absence of Inx4, Inx2 is not apically recruited, but that in such conditions Inx2 is overexpressed was not documented. Moreover, some aspects of their developmental profile (increased expression over time, change at oocyte cortex (as asked by R#2, for instance)) were not described before. Thus, our results bring new data, and most of all, this description is useful to introduce the subsequent experiments. We would therefore prefer to maintain these results in this article.

4. Figure 1G, L- Did the authors check the status of sub apical Inx2 in the follicle cells when Inx4 was depleted from the germline cells?

We apologize if our description of these results was not clear enough. Subapical/lateral localization of Inx2 was unaffected. We now state it more explicitly: “Similarly, when *Inx4* was knocked down in germline cysts, we did not detect Inx2 plaques at the germline contact, although Inx2 protein expression was increased in follicle cells <u>and still localized at the lateral domain of the cells (Fig</u>. 1G’’’)”.

5. Since the distribution of CG43693 is mostly apical and lateral in the follicle cells, it is difficult for one to understand as to how the aminoacids are internalized in the follicle cells? One would presume that uptake of amino acid may be facilitated from the basal side which is exposed to outside.

First, Coch is also present on the basal domain, though at lower level. The strongest lateral localization could reflect that there are two membranes juxtaposed there and the strongest apical one that this membrane is known to be more circumvallated, though we cannot not exclude that the apical localization of the mRNA, or another mechanism, impacts the localization of the protein.
Moreover, as we initially mentioned in the discussion, septate junctions of the follicular epithelium are not mature at these stages and small molecules can diffuse in between follicle cells and probably even between germ cells and follicle cells and thus the import can be potentially done by any domain of follicle cells. This point is now mentioned in the results to make it clearer:
The GFP decorates the whole cortex of follicle cells, though appearing more enriched at the lateral and apical domains (Fig. 3G). Of notice, septate junctions are not mature from stage 1 to 8, and the epithelium is therefore not impermeable, suggesting that solute import via Coch can occur from any domain (IsastiSanchez et al; 2021).

6. Since Inx2-Inx4 heteromeric channels have been previously demonstrated (Sahu et al 2021) to mediate calcium flow between follicle cells and nurse cells, have the authors checked if the calcium could play a role in this process?

It is indeed a possibility that we cannot currently exclude. The only compelling experimental idea we could think of is actually similar to the one we achieved by combining two binary systems and trying to rescue the loss of Inx2 in the soma by overexpressing a constitutive Ca2+ channel in the germline. This would require the generation of new transgenes, their validation with calcium sensors, etc..
Additionally, the list of ions and molecules that can pass through these junctions is likely very long and, as mentioned in our discussion, it is very likely that many amino acids or metabolites may be important for germline growth. Moreover, Sahu et al have elegantly shown that calcium transits from the germline to the soma, while in the case of germline growth it seems more likely that gap junctions are involved in a transport from the soma to the germline. So, we think that, without a more specific and presumptive link between calcium transit and growth control, developing tools to specifically test this idea is beyond the scope of our article. Nonetheless, we added a sentence in the discussion to underlie the potential role of calcium :
"Moreover, it has already been shown that calcium can be exchanged between germ cells and follicle cells by Inx2/Inx4 gap junctions, and a potential role in the control of germ cell growth of this ion that can act a second messenger cannot be currently excluded (Sahu et al, 2021)."

7. As Inx2 is known to physically associate with DE-Cadherin and Armadillo (Bauer et al 2006), it will be important for the authors to check if the structural component or channel function of the gap junction is responsible for the observed growth defect. It will be worth checking the localization of CG43693- GFP in the follicle cells in Inx2 and Inx4 depleted backgrounds.

First, as requested, we looked at Coch-GFP in *Inx2* mutant clones and observed no difference in localisation, but a slight decrease in expression level. The reason for the lower expression in unknown, but it seems unlikely that it can contribute significantly to the effect of Inx2 of germline growth. These data are no provided figure S3D and a sentence has been added in the results :
Moreover, Coch-GFP subcellular localization was not affected in mutant cells for *Inx2,* though its expression appears slightly lower in such a context (Fig. S3D)

We also attempted to provide a better demonstration of Inx2 channel activity requirement for germline growth. Interestingly, it has been developed a nice system using a RNAi transgene against Inx2 and INX2-mRFP transgenes insensitive to the RNAi with a wildtype protein sequence or containing a point mutation that is proposed to block channel activity (Miao et al, 2020, PMID: 32668209). Unfortunately, we felt to obtain a proper rescue of ovary growth defect with the WT control transgene as shown on this graph :

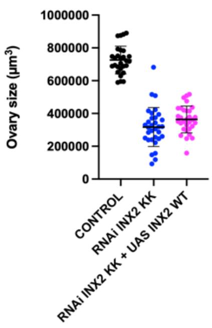

The reason for this result is unclear, a potential explanation being that the mRFP alters channel function. Indeed, to our knowledge, these transgenes have not been validated in a context where the channel function is actually needed. Thus, these experiments appeared unconclusive and were not included in the article.

8. The authors are assessing the overall size of the ovary as readout for the germline growth. Since the size of the ovary can also be regulated by the number of ovarioles, it would be best to verify the results at the level of egg chambers (stage distribution and size) (Fig 3A-B, Fig 4).

As explained to R#2 (point #1), stage distribution and egg laying quantifications are now provided (Figs 3,4, and S4). However, we did not quantified follicle size because size and stage are intrinsically linked parameters, i.e. many stages are in part defined by their size.
Also, we would like to underly that the rescue observed in figure 4 cannot be explained by a difference in gonadic development as the germline driver we used starts to be expressed in at the stages of follicle encapsulation and therefore after gonad development. This point is now mentioned in the results :
"A *QUASp:GFP* transgene driven by MatTub:QF was expressed once the germline cyst is formed in the germarium and in all the subsequent stages, starting slightly earlier than what described for MatTub:Gal4Vp16, indicating that both driver and QUASp promoter were functional (Fig. S4). …Importantly, since MatTub:QF driver is expressed only from late stages of germarium, the observed rescue is not due to an effect during gonad development. "

9. Did the authors try to rescue the germ line growth defect induced Inx2RNAi by over expressing coch in the germline itself? Something along the lines of Fig 4.

As mentioned for R#2 (point #2), we would like to also thank R#3 for this suggestion. As explained before, we found a rescue tendency both for ovary size and p-bodies. These experiments strongly argue for our general scheme.

10. The nature of the P bodies observed is different when Inx2 is down regulated in the follicle cell (Fig 5F) compared when coch RNAi is overexpressed in the follicle cell (Fig 5H). In Fig 5F the P bodies are punctate while in Fig 5H it seems more filamentous. Did the authors evaluate the P bodies when Inx4 was depleted in the germline cells.

We checked the formation of P-bodies upon *Inx4* RNAi induction in the germline and observed a very significant increase, fitting with our previous results. These data have been included into the figure 5 and the text has been modified accordingly:
"We knocked down *Inx2* and *coch* in follicle cells using tj:Gal4 in the presence of Me31B-GFP. Constitutive *Inx2* RNAi in the somatic lineage completely blocked oogenesis and precluded the observation of stages 8-9. Therefore, we added a constitutive Gal80ts transgene to allow expression of the RNAi construct only during a short period of time at the permissive temperature (14 hours, at 30°C). We also attempted *Inx4* knock-down by inducing RNAi specifically in the germline. *Coch*, *Inx2* and *Inx4* knock-downs strongly induced the formation of P-bodies when compared to their respective control, suggesting that the observed growth limitation was linked to their formation and a potential arrest of translation (Fig 5F-L).

Regarding the aspect, we agree that P-bodies may look different from a follicle to another. We also noticed it but we could not correlate it with a specific stage, food our genetic conditions. A sentence has been added to mention this point:
We also noticed that P-body aspect may differ from a follicle to another, being more dotty or more flaky, though we could not find an explanation for this difference as it appeared not linked to the genotype or the amount of P-bodies.

11. Have the authors attempted to rescue the effects of coch-RNAi by supplementing the ovaries under live culture conditions with amino acids?

It is indeed tempting to know whether *ex vivo* experiments could help to confirm our results. However, as we understand the mechanism we described, supplementing with amino acids could work if Coch was involved in amino acid synthesis but not in their import. Moreover, there is no description in the literature of a purely synthetic medium allowing to sustain follicle survival and growth and of which the composition could be modulated to compensate our mimic the effect of the loss of *coch*. Thus, with our current technical knowledge, such experiments appear impossible to realize.

Minor comments:
1. How was the follicle volume calculated? The Materials & Methods section needs to be more elaborate.

We added this sentence in the Methods :

" Mutant-control follicle volume ratios were calculated after estimating the follicle volume as a spheroid (= $4/3\pi(\text{length x width}^2)$)."

2. How do the authors arrive at the statement that "Thus, both CG43693 tissue distribution and subcellular localization were in agreement with the hypothesis that it is implicated in the import of amino acids to promote follicle growth."

We hope that our explanation provided for point #5 replies to this question. Nonetheless, we changed this sentence to make it more explicit :

Thus, both Coch tissue pattern, specific to somatic cells and present at all stages, and subcellular localization, at the cell cortex, were in agreement with the hypothesis that it is implicated in the import of amino acids to promote follicle growth.

3. Could the TOR signaling in the follicle cells affect oocyte growth in the same ways as the authors observed for Inx2?

We looked at the impact of Tor pathway on Inx2 protein expression, both in mutants blocking the pathway (Tor), or leading to its overactivity (tsc1). While Tor null mutant affect Inx2 expression, it is not the case of *tsc1* mutant. These data are now provided figure S6 and are mentioned in the text as following:

Moreover, we observed that Inx2 protein level was also sensitive to the loss but not the gain of function of the Tor pathway, suggesting that it is not equally controlled by all the pathways modulating cell growth (Figure S6C,D).
